# The Role of Nutrition in Degenerative Cervical Myelopathy: A Systematic Review

**DOI:** 10.1177/11786388211054664

**Published:** 2021-10-30

**Authors:** Celine I Partha Sarathi, Oliver D Mowforth, Amil Sinha, Faheem Bhatti, Aniqah Bhatti, Melika Akhbari, Shahzaib Ahmed, Benjamin M Davies

**Affiliations:** Division of Neurosurgery, Department of Clinical Neurosciences, Addenbrooke’s Hospital, University of Cambridge, Cambridge, UK

**Keywords:** Cervical cord, myelopathy, spondylosis, stenosis, ossification posterior longitudinal ligament, degeneration, nutritional status, malnutrition, vitamins, minerals, obesity, body weight, gastrointestinal diseases, electrolytes, hospitalisation

## Abstract

**Introduction:**

Degenerative cervical myelopathy (DCM) is the commonest cause of adult spinal cord impairment worldwide, encompassing chronic compression of the spinal cord, neurological disability and diminished quality of life. Evidence on the contribution of environmental factors is sparse; in particular, the role of nutrition in DCM is unknown. The objective of this review was to assess the effect of nutrition on DCM susceptibility, severity and surgical outcome.

**Methods:**

A systematic review in MEDLINE and Embase was conducted following PRISMA guidelines. Full-text papers in English papers, focussing on cervical myelopathy and nutrition, published before January 2020 were considered eligible. Quality assessments were performed using the GRADE assessment tool. Patient demographics, nutritional factor and DCM outcomes measures were recorded. Relationships between nutritional factors, interventions and disease prognosis were assessed.

**Results:**

In total, 5835 papers were identified of which 44 were included in the final analysis. DCM patients with pathological weight pre-operatively were more likely to see poorer improvements post-surgically. These patients experienced poorer physical and mental health improvements from surgery compared to normal weight patients and were more likely to suffer from post-operative complications such as infection, DVT, PE and hospital readmissions. Two trials reporting benefits of nutritional supplements were identified, with 1 suggesting Cerebrolysin to be significant in functional improvement. An unbalanced diet, history of alcohol abuse and malnourishment were associated with poorer post-operative outcome.

**Conclusion:**

Although the overall strength of recommendation is low, current evidence suggests nutrition may have a significant role in optimising surgical outcome in DCM patients. Although it may have a role in onset and severity of DCM, this is a preliminary suggestion. Further work needs to be done on how nutrition is defined and measured, however, the beneficial results from studies with nutritional interventions suggest nutrition could be a treatment target in DCM.

## Introduction

Degenerative cervical myelopathy (DCM) is a condition of cervical spinal cord dysfunction second to cervical stenosis from congenital and/or degenerative pathology. Degenerative processes include cervical spondylosis, ossification of the posterior longitudinal ligament, (OPLL), ossification of the ligamentum flavum and degenerative disc disease.^1^ DCM is the commonest cause of spinal cord impairment in adults worldwide.^2^

Surgical decompression is the mainstay of treatment and for most is able to halt disease progression and afford meaningful benefit.^3,4^ However, since the regenerative capacity of the spinal cord is limited, there is often permanent neurological disability^5^ and people living with DCM have amongst the poorest quality of life of any chronic disease.^6^ Therefore, offering treatment before there is permanent spinal cord damage should prevent this, but there are many uncertainties challenging such practice today.

These include the pathophysiology of DCM and the natural history. Whilst approximately 1 in 5 adults without symptoms will have evidence of spinal cord compression on their MRI due to incidental degenerative changes in the spine, it is estimated only 10% will go on to develop DCM with time.^7^ This indicates that the perception that DCM is driven uniquely by spinal cord compression is an over-simplification,^8^ and additional, dynamic factors must contribute to the aetiology of DCM, and/or influence the vulnerability of the spinal cord. Early investigations have identified candidate genetic determinants in small series,^9^ but environmental factors are also likely.

Nutrition is an important environmental factor in many diseases. Adequate nutritional status is defined as food intake sufficient to meet their requirements to support optimal health and well-being.^10^ Repair and rehabilitation requires adequate nutrition to support regenerative processes and prevent degeneration; for example, gross vitamin deficiencies can cause spinal degeneration^11,12^ and it is likely this represents one end of a spectrum.^13^ Nutritional status is also linked to recovery from surgery. Surgery itself is a significant stress that activates inflammatory and catabolic pathways; an appropriate nutritional status allows the body to respond optimally and recover efficiently,^14^ and nutritional interventions have become an important component of many enhanced recovery protocols across surgical disciplines.^15^ The topic of nutrition is also frequently raised by people with DCM, in the Myelopathy.org community and was identified as an unanswered research question by the James Lind Alliance research priority setting partnership conducted as part of AO Spine RECODE-DCM (aospine.org/recode), albeit falling outside the top 10.

Therefore, the objective of this study was to conduct the first systematic review on the role of nutrition in the onset and severity of DCM experienced, and its role in patients’ response to surgical treatment.

## Methods

A systematic review was conducted in accordance with PRISMA guidelines.^16^ A search of Embase and MEDLINE via Ovid for all papers published before 28 January 2020 was performed using previously developed search filters.^17^ The full search strategy is outlined in Supplemental Appendix 1. All primary clinical studies, available in English, considering an aspect of nutrition exclusively in the context of cervical myelopathy were considered eligible. ‘Nutrition’ encompassed all components that can be affected by diet, including: weight, electrolytes, vitamins, minerals and overall gastrointestinal (GI) health, since these can all be affected by the food one eats. If studies considered more than 1 nutrition factor, (eg, both weight and GI health) they were then assigned to all relevant categories, so that the influence of all assessed factors could be studied in depth. Animal studies, case reports, editorials, reviews, opinion articles, corrections, conference papers and studies relating to acute injury (ie, the acute stage of traumatic spinal cord injury) were excluded.

Papers were independently screened by 6 authors (CP, AS, FB, MA, AB and SA) and data were extracted (Supplemental Appendix 2). Discrepancies were settled by discussion and mutual agreement. For included studies, quality was assessed using the Grading of Recommendations, Assessment, Development and Evaluations (GRADE) assessment tool, and harvest plot made.^18^ Details of the study design, cohort demographics, intervention(s), nutrition factor(s) and outcomes were extracted.

In order to consider the differing implications of nutrition on DCM onset, severity and surgical outcomes, the reported outcomes in studies were categorised into those relating to spinal column (eg, adjacent segment disease), spinal cord (eg, neurological examination, patient-reported outcome measures, recovery-rate with treatment) and those relating to the surgical procedure (eg, adverse events, such as infection, DVT, hospital readmission). Not all studies included outcomes in all subgroups.

## Results

**Figure 1.**
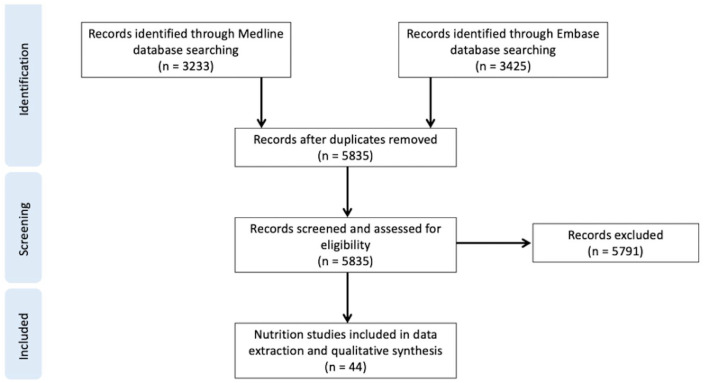
PRISMA flow diagram.^79^

A total of 6658 papers were identified by our search; 5835 remained after de-duplication. Following full-text screening, 44 were included in the final analysis. The majority (35/44 = 80%) were cohort studies (prospective and retrospective), with cohort sizes ranging from 36 to 202 694 (see Figure 1).

Based on the nutritional factor evaluated, the authors developed 4 categories to aid the collation of evidence: (1) weight, (2) malnutrition and electrolyte imbalance, (3) vitamins and minerals and (4) gastrointestinal (GI) health. Papers could be assigned to more than 1 category.

A GRADE assessment was performed to assess the quality of the papers. The GRADE score given is based on the specific outcomes measured in the papers (Supplemental Appendix 3). This data was used to formulate a harvest plot to give a visual representation of the specific outcomes assessed in individual papers (Supplemental Appendix 4). The bibliography for the harvest plot can be found in Supplemental Appendix 5.

### Weight/BMI

Both obesity and pathological weight loss are important nutrition-related factors to consider. Whilst majority of papers (21/28, 75%), found significant correlations between weight/BMI and specific DCM outcomes, a total of 7 studies (7/28, 25%) reported no significant correlation between increased weight/BMI and DCM outcome,^19-25^ For example, an international multi-centre study of 479 DCM patients reported that, although those with post-operative complications were older and had a higher BMI, these differences were statistically insignificant.^19^

#### The effect of weight on the spinal column

Weight has been shown to be a significant risk factor for developing cervical spondylosis.^20^ Higher BMI was reported to be predictive of cervical cord compression in adults with spinal deformities.^26^ One cohort study of 498 subjects reported that a high BMI significantly increased risk of cervical Modic changes (MC) in cervical spondylosis patients.^27^ Furthermore, obese patients had significantly smaller preoperative cervical lordosis, immediate postoperative lordosis and final lordosis following anterior cervical decompression and fusion ACDF^28^; the obese group experienced a significant increase in their sagittal vertical axis and a significantly decreased T1 slope during the recovery period.^28^ However, other studies reported that BMI was not a significant predictor of cervical lordosis loss after laminoplasty and that only T1 slope significantly correlates with loss of cervical lordosis.^24^

#### The effect of weight on adverse events: surgical outcomes and complications

One paper utilised a machine learning approach to report that higher weight was predictive of poorer surgical outcome,^29^ and obese patients were shown to be more likely to require surgeries of greater duration.^30^ In addition, a cohort study of 88 DCM patients demonstrated that obese patients had poorer post-operative patient-reported improvement.^31,32^ A multi-centre cohort study consisting of 8887 patients showed that obese patients had a higher risk of requiring intubation after anterior cervical surgery.^33^ Furthermore, obesity was found to be a significant predictor of 30-day hospital readmission following anterior cervical fusion surgery at 3 or more levels,^34^ although it did not significantly affect the need for revision surgery.^22^ However, a single-centre cohort study of 178 patients reported that the average BMI of patients, who did not undergo ACDF revision after initial posterior cervical fusion (PCF), was significantly higher than those who did.^35^

Elevated BMI was also significantly associated with increased risk of surgical site infections in DCM patients.^36^ Body weight was also shown to be the most important predictor in determining postoperative bleeding and venous thromboembolism in patients undergoing cervical laminoplasty.^37^ In particular, obesity in patients undergoing elective PCF has been reported to increase the risk of postoperative venous thromboembolism more than 6-fold.^38^ Elevated BMI was also shown to be associated with increased neck disability at 1-year post-surgery.^39^

On the contrary, 1 study reported that obesity was associated with a decreased risk of mortality and a higher functional independence in surgical DCM patients.^40^ Another study reported greater mental-state-improvement post-operatively in obese patients.^31^ Other studies found that both mental and physical improvements were poorer in obese patients compared to non-obese patients after anterior cervical surgery.^32,39^

In addition to obesity, weight loss has also been shown to affect surgical prognosis; ‘pathological weight loss’, defined as greater than 10% of body weight within 6 months of surgery, has been shown to be a predictor of 30-day hospital readmission in patients undergoing PCF.^41^ Similarly, pathological weight loss was found to significantly increase surgical mortality rate,^40^ and to be a significant predictor of aspiration.^42^

### Malnutrition and electrolyte imbalance

A total of 9 papers (9/44, 20%) studied the importance of malnutrition, nutritional supplementation and electrolyte imbalance in disease prognosis.

#### The effect of malnutrition and supplementation on the spinal cord

A randomised controlled trial reported that administering Cerebrolysin, a mixture of amino acids and biologically active peptides, to DCM patients who declined surgery, led to significant improvements in their myelopathy, including JOA scores and mean JOA recovery rate after 6 months.^43^ Similarly, oral creatine supplementation was shown to enhance exercise capacity in patients with cervical spinal cord injury (SCI), significantly improving oxygen uptake, carbon dioxide production and tidal volume at peak effort, with associated significantly higher peak power output; although, there was no significant effect on respiratory exchange ratio, minute ventilation and time to fatigue.^44^

#### The effect of malnutrition on adverse events: surgical outcomes and complications

Nutritional status is a significant predictor of length of hospital stay and post-operative functional motor independence.^45^ Fluid and electrolyte disturbances have been shown to be significant predictors of perioperative morbidity,^40^ post-operative aspiration^45^ and post-operative dysphagia in patients undergoing anterior cervical fusion.^46^ Furthermore, dehydration has been shown to be a significant predictor for 30-day hospital readmission after PCF.^41^

Alcohol history was also a significantly predictor of surgical outcome.^42,47^ One multi-centre consisting of 202 694 patients reported that patients with a history of alcohol abuse were at significantly higher risk of post-operative aspiration.^42^ In addition, 2 cohort studies reported that alcohol intake correlated with a delayed timing of fusion after anterior cervical surgery,^48^ and hospital readmission.^41^

In addition, a single-centre cohort study of 55 cervical myelopathy patients showed that lower prealbumin levels predicted significantly greater duration of hospital stay and higher risk of postoperative complications, including respiratory failure, skin ulcers, oesophageal perforation and infection.^49^

### Vitamins and minerals

In total, 4 papers (4/44, 9%) assessed the impact of vitamin and mineral deficiencies.

#### The effect of vitamins and minerals on spinal biology and recovery

A Brazilian multicentre cohort study reported that cobalamin, vitamin B12 and copper deficiencies were common among patients with non-traumatic myelopathy.^50^ Furthermore, a single-centre cross-sectional study, of 106 patients with chronic SCI, showed that vitamin B12 deficiency was present in 67% of patients and correlated with a longer duration of SCI.^51^ Macrocytic anaemia was present in 17% of subjects with subnormal B12 levels.^51^ Furthermore, patients with tetraplegia and tetraparesis from cervical spine pathology manifested B12 deficiency more commonly than those with paraplegia and paraparesis from thoracic or lumbar spinal pathology, however, this was not a statistically significant difference.^51^

### Gastrointestinal health

A total of 3 papers (3/44, 7%) assessed gastrointestinal (GI) health. A retrospective cohort study reported that 121 of the 757 DCM patients studied had gastrointestinal comorbidities (GIC); these patients had significantly poorer physical health scores, a higher rate of psychiatric comorbidities and were more likely to be female.^52^

#### The effect of gastrointestinal health on spinal cord biology and surgical outcome

A retrospective cohort study showed that DCM patients with GICs were significantly less neurologically impaired based on the Nurick Grade than those without GICs, although this may not be a meaningful difference. DCM patients with GICs were shown to have a lower prevalence of upper motor neurone signs and were less likely to exhibit signal intensity changes such as T1 hypointensity and T2 hyperintensity.

However, the same study reported no difference in surgical outcome between the GIC and non-GIC groups.^52^

Furthermore, a multi-centre cohort study of 479 patients reported that the presence of gastric disease was a principal component in predicting post-operative outcome in DCM patients.^53^ In particular, disability and quality of life measured by the neck disability index (NDI) and the Short Form (36) Health survey (SF-36) correlated significantly with the presence of comorbid gastric conditions.^53^

## Discussion

Nutrition, as evaluated using studies considering weight, vitamin and mineral deficiencies, malnutrition, electrolyte imbalance and gastrointestinal disease, appears to have a role in the assessment and management of DCM patients. Principally, studies were identified exploring nutrition with respect to adverse events of surgery. This association was strongest and most frequently evaluated with respect to increased BMI (obesity), describing increased risks of re-intubation,^18^ hospital readmission,^34^ bleeding,^38^ surgical site infection,^36^ venous thromboembolism^38^ and suboptimal mental recovery.^32,39^ However, studies were also identified linking nutrition to the aetiology of spinal cord compression and the response of the spinal cord to compression, including 1 study, a randomised controlled trial of Cerebrolysin, suggesting benefit for supplementation.

### Adverse events of surgery

The association between increased operative adverse events and nutrition is consistent with wider experiences of surgery.

Malnutrition is recognised as a key factor for patients undergoing surgery,^54^ linked to complications such as wound healing and surgical site infection.^55-57^ Surgery itself is a significant insult for the human body,^58^ and nutrition a fundamental component of growth and repair. This has led to initiatives such as Enhanced Recovery After Surgery (ERAS) which focus on peri-operative nutrition.^59,60^ These approaches are most prominent within gastrointestinal surgery,^61^ but increasingly applied to other specialities including spinal surgery.^62,63^ A prospective observational study in spinal surgery in the USA is on-going.^64^

Conversely, the implications of increased BMI extend beyond just nutrition. An increased BMI can create challenges throughout spinal surgical care,^65-67^ including diagnostic imaging, response to physical therapy, intubation and on-table position, surgical technique and susceptibility to peri-operative complications. Most of these challenges relate to body habitus; for example, in general positioning, or raised intrabdominal pressure increase intravenous pressure, contributing to increased operative blood loss and risk of thrombosis, however, the underlying mechanism is likely multifaceted.^38^ Establishing this is challenging as BMI frequently interacts with other significant factors such as co-morbidities. In a global study of 479 DCM patients, whilst the relationship between high BMI and post-operative complications was insignificant, comorbidities such as diabetes mellitus, cardiovascular disease and gastrointestinal disease were associated with increased post-operative complications.^19^ As identified in this review,^40^ literature synthesis across spinal surgery demonstrate that increase BMI is not consistently an unfavourable factor.^68-70^ Therefore, whilst the association is significant, the causation is less well defined.

### Spinal cord biology

The aforementioned, and well evidenced implications, for nutrition and surgery have made exploring a primary relationship between nutrition and the disease itself difficult. This is further compounded by the typical use of weight as a marker of nutrition, which is a recognised oversimplification, as even in elevated states (eg, obesity), a patient can be nutritionally deplete due to an unbalanced diet.^71^ Furthermore, spinal cord injury can result in a profound change in body composition, best characterised by a reduction in the protein content of skeletal muscle.^72^ That said, a number of studies were identified suggesting a tentative, but significant, link.

A recent linkage study, using the New York State Inpatient Data Base, compared subsequent care between morbidly obese patients that had undergone bariatric surgery and those who had not: The bariatric group, in a multi-variate model controlling for age and co-morbidities, were significantly more likely to subsequently undergo spinal surgery, in particular treatment for DCM. Amongst the author’s hypotheses for this is a consequence of post-operative malnourishment that can follow bariatric surgery.^73,74^

Other nutritional dimensions may also have a role. Nouri et al^55^ in their secondary analysis of the AO Spine series, identified a differing pattern of symptoms and assessment findings between those with and without gastro-intestinal co-morbidities. Further, there appears to be associations between spinal cord injury, deficiencies in vitamins such as vitamin B12,^51^ and vitamin D^12^ and critical subunits for neural repair, over time.^13^ Nouri et al^75^ found in a retrospective analysis of 725 patients undergoing surgery for DCM or degenerative cervical radiculopathy, that general anaemia and macrocytic anaemia (a potential consequence of B12 deficiency) was associated with poorer baseline neurological function.

Further, Allam et al (2017),^43^ conducted a randomised controlled study (*N* = 192), where patients declining surgery for DCM were given Cerebrolysin, a mixture of amino-acids and peptides given via intramuscular injection. Although, both arms improved on average without surgery (a methodological concern), this was more likely, and of greater magnitude, in the treatment arm as measured using the Japanese Orthopaedic Association score. It is important to note that despite this improvement, at their last follow-up at 6 months, patients in all groups were deteriorating from their initial improvement, suggesting that, whilst Cerebrolysin supplementation may have a significant role, its benefits may be limited to the short term.

### Limitations and future directions

This is the first systematic review to aggregate evidence relating to nutritional factors and DCM. Nutrition is a broad concept (eg, in this review, pathological weight includes both low and high BMI) and current evidence is sparse, with few studies on any 1 topic and many areas remaining completely unexplored, despite an intensive and broad search strategy. This, coupled with inconsistent data reporting,^76^ has precluded quantitative analysis, and studies are instead discussed and compared qualitatively.

Further, although the aggregated evidence relating nutrition to undergoing surgery is more consistent and well justified within the wider literature, this has made it more difficult to consider a relationship within respect to DCM onset/severity itself, as most observational studies included adverse events relating to surgical treatment. Therefore, in this study, assessments are considered by stratifying outcomes related to the surgery specifically (adverse events) from the spinal cord (neuromuscular function); however, this has limitations.

Additionally, the use of surrogates for nutrition such as weight, have inaccuracies. The measurement of nutritional status clinically is challenging.^77^ Body weight, composition or serological markers can misrepresent an individual’s status. This is a significant challenge for interventional studies within nutrition, particularly in mixed populations.^78^ Further investigation in this area will need to consider this.

Robust measures have been taken throughout this manuscript to ensure the quality of evidence is evaluated and the strength of recommendation clearly apparent (GRADE, see Supplemental Appendices 3 and 4).^18^ Therefore, whilst promising, the evidence linking nutrition to DCM are at best preliminary thus far.

However, the findings of the Cerebrolysin study, which do not include these particular limitations, presents an opportunity for nutritional interventions in DCM that will need further exploration.

## Conclusion

Nutrition, particularly weight, may have a role in the post-operative outcomes of DCM (High Strength Recommendation). Further, there is increasing evidence that nutrition, particularly supplementation, may have a role in both the onset and severity of DCM (Low Strength Recommendation). Whilst its role is likely to be multifactorial, as a modifiable factor, its better characterisation is a desirable target to support improved outcomes in the short-term. However, to truly understand its significance in DCM, future work must also consider how nutrition is defined and measured.

## Supplemental Material

sj-docx-1-nmi-10.1177_11786388211054664 – Supplemental material for The Role of Nutrition in Degenerative Cervical Myelopathy: A Systematic Review Click here for additional data file. Supplemental material, sj-docx-1-nmi-10.1177_11786388211054664 for The Role of Nutrition in Degenerative Cervical Myelopathy: A Systematic Review by Celine I Partha Sarathi, Oliver D Mowforth, Amil Sinha, Faheem Bhatti, Aniqah Bhatti, Melika Akhbari, Shahzaib Ahmed and Benjamin M Davies in Nutrition and Metabolic Insights

sj-docx-2-nmi-10.1177_11786388211054664 – Supplemental material for The Role of Nutrition in Degenerative Cervical Myelopathy: A Systematic Review Click here for additional data file. Supplemental material, sj-docx-2-nmi-10.1177_11786388211054664 for The Role of Nutrition in Degenerative Cervical Myelopathy: A Systematic Review by Celine I Partha Sarathi, Oliver D Mowforth, Amil Sinha, Faheem Bhatti, Aniqah Bhatti, Melika Akhbari, Shahzaib Ahmed and Benjamin M Davies in Nutrition and Metabolic Insights

sj-pdf-1-nmi-10.1177_11786388211054664 – Supplemental material for The Role of Nutrition in Degenerative Cervical Myelopathy: A Systematic Review Click here for additional data file. Supplemental material, sj-pdf-1-nmi-10.1177_11786388211054664 for The Role of Nutrition in Degenerative Cervical Myelopathy: A Systematic Review by Celine I Partha Sarathi, Oliver D Mowforth, Amil Sinha, Faheem Bhatti, Aniqah Bhatti, Melika Akhbari, Shahzaib Ahmed and Benjamin M Davies in Nutrition and Metabolic Insights

sj-pdf-2-nmi-10.1177_11786388211054664 – Supplemental material for The Role of Nutrition in Degenerative Cervical Myelopathy: A Systematic Review Click here for additional data file. Supplemental material, sj-pdf-2-nmi-10.1177_11786388211054664 for The Role of Nutrition in Degenerative Cervical Myelopathy: A Systematic Review by Celine I Partha Sarathi, Oliver D Mowforth, Amil Sinha, Faheem Bhatti, Aniqah Bhatti, Melika Akhbari, Shahzaib Ahmed and Benjamin M Davies in Nutrition and Metabolic Insights

sj-xlsx-1-nmi-10.1177_11786388211054664 – Supplemental material for The Role of Nutrition in Degenerative Cervical Myelopathy: A Systematic Review Click here for additional data file. Supplemental material, sj-xlsx-1-nmi-10.1177_11786388211054664 for The Role of Nutrition in Degenerative Cervical Myelopathy: A Systematic Review by Celine I Partha Sarathi, Oliver D Mowforth, Amil Sinha, Faheem Bhatti, Aniqah Bhatti, Melika Akhbari, Shahzaib Ahmed and Benjamin M Davies in Nutrition and Metabolic Insights
