# Supplementary material for: The Role of Nutrition in Degenerative Cervical Myelopathy: A Systematic Review
Source: Nutr Metab Insights. 2021 Oct 30;14:11786388211054664. doi: 10.1177/11786388211054664 (PMC8558601; doi:10.1177/11786388211054664)
Supplement: sj-docx-1-nmi-10.1177_11786388211054664 – Supplemental material for The Role of Nutrition in Degenerative Cervical Myelopathy: A Systematic Review [file sj-docx-1-nmi-10.1177_11786388211054664.docx]

| **Theme** | **Relationship examined** | **Total papers** | **Outcome measured (GRADE score)** | | |
| --- | --- | --- | --- | --- | --- |
|  |  |  | **Main adverse events** | **Spinal cord biology** | **Spinal column biology** |
| 1. **Weight (28)** | **Significant negative correlation** between **increased weight/BMI** and DCM outcome | 6  🡪  (1)(2)(3)(4)(5)(6) | Post-operative complications (⨁⨁⨁◯ MODERATE)  Mortality (⨁◯◯◯ VERY LOW) | Patient reported outcomes  (⨁⨁◯◯ LOW)  Change in Japanese Orthopaedic Association score (⨁⨁◯◯ LOW)  Functional independence measure (⨁⨁◯◯ LOW)  Reactive oxygen metabolites (⨁⨁◯◯ LOW) | Cervical alignment parameters (⨁⨁◯◯ LOW)  Adjacent segment disease (⨁⨁◯◯ LOW)  Loss of cervical lordosis (⨁⨁◯◯ LOW)  Weight as a risk factor for cervical spondylosis (⨁⨁◯◯ LOW)  Modic changes (⨁⨁◯◯ LOW) |
|  | **Significant positive correlation** between **increased weight/ BMI** and DCM outcome | 10  🡪  (7)(8)(9)(10)(11)(12)(13)(14)(1)(15) |  |  |  |
|  | **No significant correlation** between **increased weight/BMI** and DCM outcome | 9  🡪  (16)(11)(17)(5)(18)(19)(20)(21)(22) |  |  |  |
|  | **Significant negative correlation** between **pre-operative weight loss** and DCM outcome | 0 |  |  |  |
|  | **Significant positive correlation** between **pre-operative weight loss** and DCM outcome | 4  🡪  (4)(23)(24)(25) |  |  |  |
|  | **No significant correlation** between **pre-operative weight loss** and DCM outcome | 0 |  |  |  |
| 1. **Malnutrition & Electrolyte Imbalance (9)** | **Significant negative correlation** between **malnutrition/ electrolyte** imbalance and DCM outcome | 2  🡪  (14)(26) | Post-operative complications (⨁⨁⨁◯ MODERATE)  Timing of fusion (⨁⨁◯◯ LOW) | Functional independence measure efficiency (⨁⨁◯◯ LOW)  Change in the Japanese Orthopaedic Association score (RCT) (⨁⨁⨁⨁ HIGH) |  |
|  | **Significant positive correlation** between **malnutrition/ electrolyte** imbalance and DCM outcome | 7  🡪  (4)(25)(23)(24)(27)(28)(29) |  |  |  |
|  | **No significant correlation** between **malnutrition/ electrolyte** imbalance and DCM outcome | 1  🡪  (29) |  |  |  |
| 1. **Vitamins & Minerals (4)** | **Significant negative correlation** between **vitamin/mineral status** and DCM outcome | 1  🡪  (30) |  | Upper extremity work capacity (⨁⨁⨁⨁ HIGH)  Change in Japanese Orthopaedic Association score (RCT) (⨁⨁⨁⨁ HIGH)  Vitamin D deficiency (⨁⨁◯◯ LOW) | OPLL (⨁⨁◯◯ LOW) |
|  | **Significant positive correlation** between **vitamin/mineral status** and DCM outcome | 3  🡪  (26)(32)(33) |  |  |  |
|  | **No significant correlation** between **vitamin/mineral status** and DCM outcome | 0 |  |  |  |
| 1. **Gastrointestinal Health (3)** | **Significant negative correlation** between **gastrointestinal health status** and DCM outcome | 2  🡪  (34)(35) | Post-operative complications (⨁⨁◯◯ LOW) | Change in Japanese Orthopaedic Association score (⨁⨁◯◯ LOW)  Patient reported outcomes (⨁⨁◯◯ LOW) |  |
|  | **Significant positive correlation** between **gastrointestinal health status** and DCM outcome | 1  🡪  (12) |  |  |  |
|  | **No significant correlation** between **gastrointestinal health status** and DCM outcome | 1  🡪  (34) |  |  |  |

**Quality Assessment: GRADE Table**

Papers assessing the impact of a particular theme (e.g. weight) on various, specific DCM outcomes (e.g. post-operative complications) were included and assessed using the standardised GRADE framework. This was done using the GRADEpro GDT software. (36) A final grade for that specific DCM outcome (e.g. ⨁⨁⨁⨁ HIGH) was calculated to represent the overall quality of studies. These outcomes were then grouped into the broader categories of adverse events, spinal cord biology, spinal column biology and other. For clarity, below are the criteria and methods used by studies to describe the more specific outcomes:

**Definitions**

- Post-operative complications – The complications measured in papers included under this outcome consist of: concurrent cervical spinal cord compression, superficial site infections, deep site infections (fascial and muscle layers), organ or space site infections (any location apart from the operational incision), reintubation, anterior haematoma evacuation, spinal epidural haematoma, pseudoarthrosis, hardware failure, screw malposition, C5 radiculopathy, axial pain, new intractable neck pain, adjacent segment degeneration, instability, dural tear, neurological deterioration, progression of myelopathy, cortical blindness, non-union, graft dislodgment/migration, graft site pain, post-operative kyphosis, cardiopulmonary event, stroke, deep vein thrombosis, venous thromboembolism, renal, peripheral nerve injury, blood transfusions required, urinary tract infections, wound complications, aspiration and acute respiratory distress syndrome. (7)(11)(28)(8)(9)(10)(12)(4)(23)(27)(29)(2)(16)(13)(24)(25)
- Patient reported outcomes – Papers included under this outcome used the following patient questionnaires and scoring systems: the neck disability index (NDI – assesses how neck pain is impacting daily life), short form 36 health survey (SF-36 – measures the impact of clinical interventions on daily life using both physical and mental components) and visual analogue scale scores for neck and arm pain. (1)(3)(5)(6)(17)(34)(35)
- Functional independence measure efficiency (FIM efficiency) – FIM measures individual’s physical, psychological and social function using the level of assistance an individual needs, and their functional status is graded from total independence to total assistance. FIM efficiency is the change in FIM score per day and is calculated by change in FIM score between admission and discharge divided by the length of stay. (14)

1. Basques BA, Khan JM, Louie PK, Mormol J, Heidt S, Varthi A, et al. Obesity does not impact clinical outcome but affects cervical sagittal alignment and adjacent segment degeneration in short term follow-up after an anterior cervical decompression and fusion. Spine J Off J North Am Spine Soc. 2019;19(7):1146–53.

2. Wang TY, Lubelski D, Abdullah KG, Steinmetz MP, Benzel EC, Mroz TE. Rates of anterior cervical discectomy and fusion after initial posterior cervical foraminotomy. Spine J Off J North Am Spine Soc. 2015 May 1;15(5):971–6.

3. Merali ZG, Witiw CD, Badhiwala JH, Wilson JR, Fehlings MG. Using a machine learning approach to predict outcome after surgery for degenerative cervical myelopathy. PloS One. 2019;14(4):e0215133.

4. Kaye ID, Marascalchi BJ, Macagno AE, Lafage VA, Bendo JA, Passias PG. Predictors of morbidity and mortality among patients with cervical spondylotic myelopathy treated surgically. Eur Spine J Off Publ Eur Spine Soc Eur Spinal Deform Soc Eur Sect Cerv Spine Res Soc. 2015 Dec;24(12):2910–7.

5. Wilson JR, Tetreault LA, Schroeder G, Harrop JS, Prasad S, Vaccaro A, et al. Impact of Elevated Body Mass Index and Obesity on Long-term Surgical Outcomes for Patients With Degenerative Cervical Myelopathy: Analysis of a Combined Prospective Dataset. Spine. 2017 Feb;42(3):195–201.

6. Auffinger B, Lam S, Kraninger J, Shen J, Roitberg BZ. The impact of obesity on surgeon ratings and patient-reported outcome measures after degenerative cervical spine disease surgery. World Neurosurg. 2014 Aug;82(1–2):e345-352.

7. Shimizu T, Lehman RA, Pongmanee S, Alex Sielatycki J, Leung E, Riew KD, et al. Prevalence and Predictive Factors of Concurrent Cervical Spinal Cord Compression in Adult Spinal Deformity. Spine. 2019 Aug 1;44(15):1049–56.

8. Jalai CM, Worley N, Poorman GW, Cruz DL, Vira S, Passias PG. Surgical site infections following operative management of cervical spondylotic myelopathy: prevalence, predictors of occurence, and influence on peri-operative outcomes. Eur Spine J Off Publ Eur Spine Soc Eur Spinal Deform Soc Eur Sect Cerv Spine Res Soc. 2016;25(6):1891–6.

9. Nagoshi N, Fehlings MG, Nakashima H, Tetreault L, Gum JL, Smith ZA, et al. Prevalence and Outcomes in Patients Undergoing Reintubation After Anterior Cervical Spine Surgery: Results From the AOSpine North America Multicenter Study on 8887 Patients. Glob Spine J. 2017 Apr;7(1 Suppl):96S-102S.

10. Yamada K, Abe Y, Satoh S, Yanagibashi Y, Hyakumachi T, Masuda T. Large Increase in Blood Pressure After Extubation and High Body Mass Index Elevate the Risk of Spinal Epidural Hematoma After Spinal Surgery. Spine. 2015 Jul 1;40(13):1046–52.

11. Phan K, Kothari P, Lee NJ, Virk S, Kim JS, Cho SK. Impact of Obesity on Outcomes in Adults Undergoing Elective Posterior Cervical Fusion. Spine. 2017 Feb 15;42(4):261–6.

12. Tetreault L, Tan G, Kopjar B, Côté P, Arnold P, Nugaeva N, et al. Clinical and Surgical Predictors of Complications Following Surgery for the Treatment of Cervical Spondylotic Myelopathy: Results From the Multicenter, Prospective AOSpine International Study of 479 Patients. Neurosurgery. 2016 Jul;79(1):33–44.

13. Puvanesarajah V, Hassanzadeh H, Shimer AL, Shen FH, Singla A. Readmission Rates, Reasons, and Risk Factors Following Anterior Cervical Fusion for Cervical Spondylosis in Patients Above 65 Years of Age. Spine. 2017 Jan 15;42(2):78–84.

14. Tanaka M, Momosaki R, Wakabayashi H, Kikura T, Maeda K. Relationship between nutritional status and improved ADL in individuals with cervical spinal cord injury in a convalescent rehabilitation ward. Spinal Cord. 2019 Jun;57(6):501–8.

15. Bai J, Yu K, Sun Y, Kong L, Shen Y. Prevalence of and risk factors for Modic change in patients with symptomatic cervical spondylosis: an observational study. J Pain Res. 2018 Feb 14;11:355–60.

16. van Eck CF, Regan C, Donaldson WF, Kang JD, Lee JY. The revision rate and occurrence of adjacent segment disease after anterior cervical discectomy and fusion: a study of 672 consecutive patients. Spine. 2014 Dec 15;39(26):2143–7.

17. Sielatycki JA, Chotai S, Kay H, Stonko D, McGirt M, Devin CJ. Does Obesity Correlate With Worse Patient-Reported Outcomes Following Elective Anterior Cervical Discectomy and Fusion? Neurosurgery. 2016 Jul;79(1):69–74.

18. Zhang JT, Meng FT, Wang S, Wang LF, Shen Y. Predictors of surgical outcome in cervical spondylotic myelopathy: focusing on the quantitative signal intensity. Eur Spine J Off Publ Eur Spine Soc Eur Spinal Deform Soc Eur Sect Cerv Spine Res Soc. 2015 Dec;24(12):2941–5.

19. Takahashi H, Aoki Y, Saito J, Nakajima A, Sonobe M, Akatsu Y, et al. Serum oxidative stress influences neurological recovery after surgery to treat acutely worsening symptoms of compression myelopathy: a cross-sectional human study. BMC Musculoskelet Disord. 2019 Dec 7;20(1):589.

20. Zhang JT, Li JQ, Niu RJ, Liu Z, Tong T, Shen Y. Predictors of cervical lordosis loss after laminoplasty in patients with cervical spondylotic myelopathy. Eur Spine J Off Publ Eur Spine Soc Eur Spinal Deform Soc Eur Sect Cerv Spine Res Soc. 2017;26(4):1205–10.

21. You J, Tang X, Gao W, Shen Y, Ding W-Y, Ren B. Factors predicting adjacent segment disease after anterior cervical discectomy and fusion treating cervical spondylotic myelopathy. Medicine (Baltimore) [Internet]. 2018 Oct 26 [cited 2020 May 5];97(43). Available from: https://www.ncbi.nlm.nih.gov/pmc/articles/PMC6221637/

22. Singh S, Kumar D, Kumar S. Risk factors in cervical spondylosis. J Clin Orthop Trauma. 2014 Dec;5(4):221–6.

23. Fineberg SJ, Oglesby M, Patel AA, Singh K. Incidence, risk factors, and mortality associated with aspiration in cervical spine surgery. Spine. 2013 Sep 1;38(19):E1189-1195.

24. Choy W, Lam SK, Smith ZA, Dahdaleh NS. Predictors of 30-Day Hospital Readmission After Posterior Cervical Fusion in 3401 Patients. Spine. 2018 01;43(5):356–63.

25. Singh K, Marquez-Lara A, Nandyala SV, Patel AA, Fineberg SJ. Incidence and risk factors for dysphagia after anterior cervical fusion. Spine. 2013 Oct 1;38(21):1820–5.

26. Allam AFA, Abotakia TAA, Koptan W. Role of Cerebrolysin in cervical spondylotic myelopathy patients: a prospective randomized study. Spine J Off J North Am Spine Soc. 2018;18(7):1136–42.

27. Guan J, Holland CM, Ravindra VM, Bisson EF. Perioperative malnutrition and its relationship to length of stay and complications in patients undergoing surgery for cervical myelopathy. Surg Neurol Int. 2017;8:307.

28. Passias PG, Jalai CM, Worley N, Vira S, Hasan S, Horn SR, et al. Predictors of Hospital Length of Stay and 30-Day Readmission in Cervical Spondylotic Myelopathy Patients: An Analysis of 3057 Patients Using the ACS-NSQIP Database. World Neurosurg. 2018 Feb;110:e450–8.

29. Yeung KKL, Cheung PWH, Cheung JPY. Anterior cervical discectomy and fusion for cervical myelopathy using stand-alone tricortical iliac crest autograft: Predictive factors for neurological and fusion outcomes. J Orthop Surg Hong Kong. 2019 Dec;27(3):2309499019869166.

30. Kobashi G, Ohta K, Washio M, Okamoto K, Sasaki S, Yokoyama T, et al. FokI variant of vitamin D receptor gene and factors related to atherosclerosis associated with ossification of the posterior longitudinal ligament of the spine: a multi-hospital case-control study. Spine. 2008 Jul 15;33(16):E553-558.

31. Bauman WA, Zhong YG, Schwartz E. Vitamin D deficiency in veterans with chronic spinal cord injury. Metabolism. 1995 Dec;44(12):1612–6.

32. Jacobs PL, Mahoney ET, Cohn KA, Sheradsky LF, Green BA. Oral creatine supplementation enhances upper extremity work capacity in persons with cervical-level spinal cord injury. Arch Phys Med Rehabil. 2002 Jan;83(1):19–23.

33. Petchkrua W, Burns SP, Stiens SA, James JJ, Little JW. Prevalence of vitamin B12 deficiency in spinal cord injury. Arch Phys Med Rehabil. 2003 Nov;84(11):1675–9.

34. Nouri A, Badhiwala JH, Kato S, Reihani-Kermani H, Patel K, Wilson JR, et al. The Relationship Between Gastrointestinal Comorbidities, Clinical Presentation and Surgical Outcome in Patients with DCM: Analysis of a Global Cohort. J Clin Med. 2020 Feb 26;9(3).

35. Badhiwala JH, Witiw CD, Nassiri F, Jaja BNR, Akbar MA, Mansouri A, et al. Patient phenotypes associated with outcome following surgery for mild degenerative cervical myelopathy: a principal component regression analysis. Spine J Off J North Am Spine Soc. 2018;18(12):2220–31.

36. GRADEpro GDT: GRADEpro Guideline Development Tool [Software]. McMaster University, 2020 (developed by Evidence Prime, Inc.). Available from gradepro.org.
