# Supplementary material for: The Role of Nutrition in Degenerative Cervical Myelopathy: A Systematic Review
Source: Nutr Metab Insights. 2021 Oct 30;14:11786388211054664. doi: 10.1177/11786388211054664 (PMC8558601; doi:10.1177/11786388211054664)
Supplement: sj-docx-2-nmi-10.1177_11786388211054664 – Supplemental material for The Role of Nutrition in Degenerative Cervical Myelopathy: A Systematic Review [file sj-docx-2-nmi-10.1177_11786388211054664.docx]

S5. Appendix 5. Nutrition Harvest Plot Bibliography

1. Basques BA, Khan JM, Louie PK, Mormol J, Heidt S, Varthi A, et al. Obesity does not impact clinical outcome but affects cervical sagittal alignment and adjacent segment degeneration in short term follow-up after an anterior cervical decompression and fusion. Spine J Off J North Am Spine Soc. 2019;19(7):1146–53.

2. Choy W, Lam SK, Smith ZA, Dahdaleh NS. Predictors of 30-Day Hospital Readmission After Posterior Cervical Fusion in 3401 Patients. Spine. 2018 01;43(5):356–63.

3. Shimizu T, Lehman RA, Pongmanee S, Alex Sielatycki J, Leung E, Riew KD, et al. Prevalence and Predictive Factors of Concurrent Cervical Spinal Cord Compression in Adult Spinal Deformity. Spine. 2019 Aug 1;44(15):1049–56.

4. Singh K, Marquez-Lara A, Nandyala SV, Patel AA, Fineberg SJ. Incidence and risk factors for dysphagia after anterior cervical fusion. Spine. 2013 Oct 1;38(21):1820–5.

5. Fineberg SJ, Oglesby M, Patel AA, Singh K. Incidence, risk factors, and mortality associated with aspiration in cervical spine surgery. Spine. 2013 Sep 1;38(19):E1189-1195.

6. Jalai CM, Worley N, Poorman GW, Cruz DL, Vira S, Passias PG. Surgical site infections following operative management of cervical spondylotic myelopathy: prevalence, predictors of occurence, and influence on peri-operative outcomes. Eur Spine J Off Publ Eur Spine Soc Eur Spinal Deform Soc Eur Sect Cerv Spine Res Soc. 2016;25(6):1891–6.

7. Merali ZG, Witiw CD, Badhiwala JH, Wilson JR, Fehlings MG. Using a machine learning approach to predict outcome after surgery for degenerative cervical myelopathy. PloS One. 2019;14(4):e0215133.

8. Nagoshi N, Fehlings MG, Nakashima H, Tetreault L, Gum JL, Smith ZA, et al. Prevalence and Outcomes in Patients Undergoing Reintubation After Anterior Cervical Spine Surgery: Results From the AOSpine North America Multicenter Study on 8887 Patients. Glob Spine J. 2017 Apr;7(1 Suppl):96S-102S.

9. Puvanesarajah V, Hassanzadeh H, Shimer AL, Shen FH, Singla A. Readmission Rates, Reasons, and Risk Factors Following Anterior Cervical Fusion for Cervical Spondylosis in Patients Above 65 Years of Age. Spine. 2017 Jan 15;42(2):78–84.

10. Sielatycki JA, Chotai S, Kay H, Stonko D, McGirt M, Devin CJ. Does Obesity Correlate With Worse Patient-Reported Outcomes Following Elective Anterior Cervical Discectomy and Fusion? Neurosurgery. 2016 Jul;79(1):69–74.

11. Tanaka M, Momosaki R, Wakabayashi H, Kikura T, Maeda K. Relationship between nutritional status and improved ADL in individuals with cervical spinal cord injury in a convalescent rehabilitation ward. Spinal Cord. 2019 Jun;57(6):501–8.

12. Wang TY, Lubelski D, Abdullah KG, Steinmetz MP, Benzel EC, Mroz TE. Rates of anterior cervical discectomy and fusion after initial posterior cervical foraminotomy. Spine J Off J North Am Spine Soc. 2015 May 1;15(5):971–6.

13. Wilson JR, Tetreault LA, Schroeder G, Harrop JS, Prasad S, Vaccaro A, et al. Impact of Elevated Body Mass Index and Obesity on Long-term Surgical Outcomes for Patients With Degenerative Cervical Myelopathy: Analysis of a Combined Prospective Dataset. Spine. 2017 Feb;42(3):195–201.

14. Yamada K, Abe Y, Satoh S, Yanagibashi Y, Hyakumachi T, Masuda T. Large Increase in Blood Pressure After Extubation and High Body Mass Index Elevate the Risk of Spinal Epidural Hematoma After Spinal Surgery. Spine. 2015 Jul 1;40(13):1046–52.

15. Bai J, Yu K, Sun Y, Kong L, Shen Y. Prevalence of and risk factors for Modic change in patients with symptomatic cervical spondylosis: an observational study. J Pain Res. 2018;11:355–60.

16. Phan K, Kothari P, Lee NJ, Virk S, Kim JS, Cho SK. Impact of Obesity on Outcomes in Adults Undergoing Elective Posterior Cervical Fusion. Spine. 2017 Feb 15;42(4):261–6.

17. Kaye ID, Marascalchi BJ, Macagno AE, Lafage VA, Bendo JA, Passias PG. Predictors of morbidity and mortality among patients with cervical spondylotic myelopathy treated surgically. Eur Spine J Off Publ Eur Spine Soc Eur Spinal Deform Soc Eur Sect Cerv Spine Res Soc. 2015 Dec;24(12):2910–7.

18. Auffinger B, Lam S, Kraninger J, Shen J, Roitberg BZ. The impact of obesity on surgeon ratings and patient-reported outcome measures after degenerative cervical spine disease surgery. World Neurosurg. 2014 Aug;82(1–2):e345-352.

19. Tetreault L, Tan G, Kopjar B, Côté P, Arnold P, Nugaeva N, et al. Clinical and Surgical Predictors of Complications Following Surgery for the Treatment of Cervical Spondylotic Myelopathy: Results From the Multicenter, Prospective AOSpine International Study of 479 Patients. Neurosurgery. 2016 Jul;79(1):33–44.

20. Singh S, Kumar D, Kumar S. Risk factors in cervical spondylosis. J Clin Orthop Trauma. 2014 Dec;5(4):221–6.

21. Takahashi H, Aoki Y, Saito J, Nakajima A, Sonobe M, Akatsu Y, et al. Serum oxidative stress influences neurological recovery after surgery to treat acutely worsening symptoms of compression myelopathy: a cross-sectional human study. BMC Musculoskelet Disord. 2019 Dec 7;20(1):589.

22. van Eck CF, Regan C, Donaldson WF, Kang JD, Lee JY. The revision rate and occurrence of adjacent segment disease after anterior cervical discectomy and fusion: a study of 672 consecutive patients. Spine. 2014 Dec 15;39(26):2143–7.

23. You J, Tang X, Gao W, Shen Y, Ding W-Y, Ren B. Factors predicting adjacent segment disease after anterior cervical discectomy and fusion treating cervical spondylotic myelopathy. Medicine (Baltimore) [Internet]. 2018 Oct 26 [cited 2020 May 5];97(43). Available from: https://www.ncbi.nlm.nih.gov/pmc/articles/PMC6221637/

24. Zhang JT, Li JQ, Niu RJ, Liu Z, Tong T, Shen Y. Predictors of cervical lordosis loss after laminoplasty in patients with cervical spondylotic myelopathy. Eur Spine J Off Publ Eur Spine Soc Eur Spinal Deform Soc Eur Sect Cerv Spine Res Soc. 2017;26(4):1205–10.

25. Zhang JT, Meng FT, Wang S, Wang LF, Shen Y. Predictors of surgical outcome in cervical spondylotic myelopathy: focusing on the quantitative signal intensity. Eur Spine J Off Publ Eur Spine Soc Eur Spinal Deform Soc Eur Sect Cerv Spine Res Soc. 2015 Dec;24(12):2941–5.

26. Choy W, Lam SK, Smith ZA, Dahdaleh NS. Predictors of 30-Day Hospital Readmission After Posterior Cervical Fusion in 3401 Patients. Spine. 2018 01;43(5):356–63.

27. Kaye ID, Marascalchi BJ, Macagno AE, Lafage VA, Bendo JA, Passias PG. Predictors of morbidity and mortality among patients with cervical spondylotic myelopathy treated surgically. Eur Spine J Off Publ Eur Spine Soc Eur Spinal Deform Soc Eur Sect Cerv Spine Res Soc. 2015 Dec;24(12):2910–7.

28. Singh K, Marquez-Lara A, Nandyala SV, Patel AA, Fineberg SJ. Incidence and risk factors for dysphagia after anterior cervical fusion. Spine. 2013 Oct 1;38(21):1820–5.

29. Fineberg SJ, Oglesby M, Patel AA, Singh K. Incidence, risk factors, and mortality associated with aspiration in cervical spine surgery. Spine. 2013 Sep 1;38(19):E1189-1195.

30. Guan J, Holland CM, Ravindra VM, Bisson EF. Perioperative malnutrition and its relationship to length of stay and complications in patients undergoing surgery for cervical myelopathy. Surg Neurol Int. 2017;8:307.

31. Tanaka M, Momosaki R, Wakabayashi H, Kikura T, Maeda K. Relationship between nutritional status and improved ADL in individuals with cervical spinal cord injury in a convalescent rehabilitation ward. Spinal Cord. 2019 Jun;57(6):501–8.

32. Passias PG, Jalai CM, Worley N, Vira S, Hasan S, Horn SR, et al. Predictors of Hospital Length of Stay and 30-Day Readmission in Cervical Spondylotic Myelopathy Patients: An Analysis of 3057 Patients Using the ACS-NSQIP Database. World Neurosurg. 2018 Feb;110:e450–8.

33. Allam AFA, Abotakia TAA, Koptan W. Role of Cerebrolysin in cervical spondylotic myelopathy patients: a prospective randomized study. Spine J Off J North Am Spine Soc. 2018;18(7):1136–42.

34. Yeung KKL, Cheung PWH, Cheung JPY. Anterior cervical discectomy and fusion for cervical myelopathy using stand-alone tricortical iliac crest autograft: Predictive factors for neurological and fusion outcomes. J Orthop Surg Hong Kong. 2019 Dec;27(3):2309499019869166.

35. Allam AFA, Abotakia TAA, Koptan W. Role of Cerebrolysin in cervical spondylotic myelopathy patients: a prospective randomized study. Spine J Off J North Am Spine Soc. 2018;18(7):1136–42.

36. Kobashi G, Ohta K, Washio M, Okamoto K, Sasaki S, Yokoyama T, et al. FokI variant of vitamin D receptor gene and factors related to atherosclerosis associated with ossification of the posterior longitudinal ligament of the spine: a multi-hospital case-control study. Spine. 2008 Jul 15;33(16):E553-558.

37. Jacobs PL, Mahoney ET, Cohn KA, Sheradsky LF, Green BA. Oral creatine supplementation enhances upper extremity work capacity in persons with cervical-level spinal cord injury. Arch Phys Med Rehabil. 2002 Jan;83(1):19–23.

38. Petchkrua W, Burns SP, Stiens SA, James JJ, Little JW. Prevalence of vitamin B12 deficiency in spinal cord injury. Arch Phys Med Rehabil. 2003 Nov;84(11):1675–9.

39. Tetreault L, Tan G, Kopjar B, Côté P, Arnold P, Nugaeva N, et al. Clinical and Surgical Predictors of Complications Following Surgery for the Treatment of Cervical Spondylotic Myelopathy: Results From the Multicenter, Prospective AOSpine International Study of 479 Patients. Neurosurgery. 2016 Jul;79(1):33–44.

40. Nouri A, Badhiwala JH, Kato S, Reihani-Kermani H, Patel K, Wilson JR, et al. The Relationship Between Gastrointestinal Comorbidities, Clinical Presentation and Surgical Outcome in Patients with DCM: Analysis of a Global Cohort. J Clin Med. 2020 Feb 26;9(3).

41. Badhiwala JH, Witiw CD, Nassiri F, Jaja BNR, Akbar MA, Mansouri A, et al. Patient phenotypes associated with outcome following surgery for mild degenerative cervical myelopathy: a principal component regression analysis. Spine J Off J North Am Spine Soc. 2018;18(12):2220–31.
