# Supplementary material for: The Role of Nutrition in Degenerative Cervical Myelopathy: A Systematic Review
Source: Nutr Metab Insights. 2021 Oct 30;14:11786388211054664. doi: 10.1177/11786388211054664 (PMC8558601; doi:10.1177/11786388211054664)
Supplement: sj-pdf-1-nmi-10.1177_11786388211054664 – Supplemental material for The Role of Nutrition in Degenerative Cervical Myelopathy: A Systematic Review [file sj-pdf-1-nmi-10.1177_11786388211054664.pdf]

S1. Appendix 1. Search Strategy

A search strategy was devised to obtain as many hits as possible for the title, 'Nutrition in Degenerative cervical myelopathy'

POPULATION:

#	MEDLINE (from Davies, 2018)	Hits	EMBASE 1980-2020 week 4	Hits
1	exp Cervical Vertebrae/ or exp Cervical Cord/ or cervical.mp. or (phrenic nucleus or accessory nucleus).mp. or (("Japanese Orthop?edic Association" adj2 score*) or (joa adj2 score*)).mp.	257565	exp *cervical spinal cord/ or cervical.ti,ab. or exp *cervical spine/ or exp *cervical vertebra/ or (phrenic nucleus or accessory nucleus).ti,ab. or exp *Japanese Orthopaedic Association score/ or Japanese Orthop?edic Association.ti,ab. or ("Japanese Orthop?edic Association" adj2 scor*).ti,ab. or (joa adj2 scor*).ti,ab.	249509
2	myelopath*.mp. or exp Spinal Cord Diseases/ or (spinal cord adj3 (diseas* or disorder*)).mp. or myeloradiculopath*.mp. or spondylomyelopath*.mp. or spondylomyeloradiculopath*.mp. or (Spinal Cord adj3 Compress*).mp. or exp Spinal Cord Compression/	139719	myelopath*.ti,ab. or exp cervical myelopathy/ or exp *cervical spondylotic myelopathy/ or spondylotic cervical myelopathy.mp. or exp *spinal cord disease/ or "cervical spinal cord injury".ti,ab. or exp *myelography/ or exp *myeloradiculopathy/ or myeloradiculopath*.ti,ab. or exp *cervical spondylosis/ or (spinal cord adj3 (diseas* or disorder*)).ti,ab. or spondylomyelopath*.ti,ab. or (Spinal Cord adj3 Compress*).ti,ab. or exp *spinal cord compression/	151542
3	1 and 2	19050	1 and 2	20060
4	exp "Ossification of Posterior Longitudinal Ligament"/	934	"ossification of posterior longitudinal ligament".ti,ab. or exp *ligament calcinosis/ or (exp *posterior longitudinal ligament/ and (exp *ossification/ or ossifi*.ti,ab.))	1397
5	3 or 4	19518	3 or 4	20898
6	exp Atlanto-Occipital Joint/ or exp Arteriovenous Fistula/ or exp Radiotherapy/ or exp Vitamin B 12/ or exp Radiation/ or exp Radiation Injuries/ or exp Re-Irradiation/ or exp Craniospinal Irradiation/ or exp Whole-Body Irradiation/ or exp Motor Neuron Disease/ or exp Amyotrophic Lateral Sclerosis/ or exp Neoplasm Metastasis/ or exp Hemangioma/ or exp neoplasm/ or exp metastasis/ or exp Nervous System Malformations/ or exp "autoimmune diseases of the nervous system"/ or exp "congenital, hereditary, and neonatal diseases and abnormalities"/ or exp virus diseases/	5670902	exp atlantooccipital joint/ or exp arteriovenous fistula/ or exp radiotherapy/ or exp cyanocobalamin/ or exp radiation injury repair/ or exp radiation injury/ or exp *radiation/ or exp re-irradiation/ or exp irradiation/ or exp craniospinal irradiation/ or exp whole body radiation/ or exp *motor neuron disease/ or exp *amyotrophic lateral sclerosis/ or neoplasm metastasis.mp. or exp metastasis/ or exp *neoplasm/ or exp malignant neoplasm/ or exp radiation induced neoplasm/ or exp myeloproliferative neoplasm/ or exp vertebra hemangioma/ or exp hemangioma/ or exp nervous system malformation/ or autoimmune diseases of the nervous system.mp. or autoimmune nervous system.mp. or (congenital, hereditary, and neonatal diseases and abnormalities).mp. or "congenital disorder".mp. or exp genetic disorder/ or "newborn disease".mp. or exp virus infection/	5801757
7	5 not 6	14368	5 not 6	16462

S1. Appendix 1. Search Strategy

NB spondylomyeloradiculopath*.mp. does not exist in EMBASE

AND

S1. Appendix 1. Search Strategy

INTERVENTION:

#	MEDLINE	Hits	EMBASE	Hits
1	Exp food/ or food.mp or exp eating/ or exp carbohydrates/ or carbohydrate.mp or exp meat/ or exp meat products/ or exp meat proteins/ or exp red meat/ or meat.mp or exp vegans/ or vegan.mp or exp vegetarians/ or vegetarian.mp or exp diet/ or diet.mp or exp diet, carbohydrate-restricted/ or exp diet, carbohydrate loading/ or exp diet, fat-restricted/ or exp diet, gluten-free/ or exp diet, high fat/ or exp diet,, high protein/ or exp diet, ketogenic/ or exp diet, Mediterranean/ or exp diet, protein-restricted/ or exp diet, vegan/ or exp diet, vegetarian/ or exp diet, Western/ or exp diet, diabetic/ or exp diet, paleolithic/ or exp fish proteins/ or exp fish proteins, dietary/ or exp egg proteins/ or exp dietary proteins/ or exp proteins/ or exp pea proteins/ or exp whey proteins/ or exp exp healthy diet/ or exp diet, macrobiotic/ or exp dietary supplements/ or supplements.mp or exp enteral nutrition/ or feeding.mp or exp water/ or water.mp or exp body water/ or exp water deprivation/ or nutrition*.mp or exp enteral nutrition/ or exp parenteral nutrition/ or exp malnutrition/ or malnutrition.mp or exp vitamins/ or exp vitamin D/ or exp vitamin D deficiency/ or exp vitamin E/ or exp ascorbic acid/ or exp vitamin B 12/ or exp vitamin B 12 deficiency/ or exp vitamin B complex/ or vitamin.mp or cobalamin C.mp or cyanocobalamin.mp or an?emi*.mp or exp anemia/ or exp anemia, iron-deficiency/	9419039	exp *food/ or food.ti,ab or exp *food intake/ or exp *carbohydrate/ or carbohydrate.ti,ab or exp *meat/ or exp *meat protein/ or exp *red meat/ or exp *white meat/ or exp *vegan/ or exp *vegetarian/ or vegan.ti,ab or meat.ti,ab or vegetarian.ti,ab or exp *diet/ or exp *artificial diet/ or exp *diabetic diet/ or exp *diet induced obesity/ or exp *diet restriction/ or exp *diet supplementation/ or exp *fiber free diet/ or exp *fruitarian diet/ or exp *gluten free diet/ or exp *gluten free casein free diet/ or exp *healthy diet/ or exp *high calorie diet/ or exp *high fiber diet/ or exp *high glycemic index diet/ or exp *ketogenic diet/ or exp *lactose free diet/ or exp *lipid diet/ or exp *liquid diet or exp *low glycemic index diet/ or exp *macrobiotic diet/ or exp *Mediterranean diet/ or exp *obesogenic diet or exp *paleolithic diet/ or exp *pescovegetarian diet/ or exp *protein diet/ or exp *raw food diet/ or exp *renal diet/ or exp *unhealthy diet/ or exp *vegan diet/ or exp *vegetarian diet/ or exp *lactovegetarian diet/ or exp *Western diet/ or diet.ti,ab or supplement.ti,ab or exp *feeding/ or exp *feeding tube/ or exp *intravenous feeding or exp *enteric feeding/ or feeding.ti,ab or exp *water/ or water.ti,ab or nutrition*.ti,ab or exp *nutrition/ or exp *nutrition supplement/ or exp *enteral nutrition/ or exp *parenteral nutrition/ or exp *malnutrition/ or malnutrition.ti,ab or exp *protein calorie malnutrition or exp fetal malnutrition or exp *vitamin/ or exp *vitamin B complex/ or exp *vitamin D/ or exp vitamin intake/ or exp *vitamin deficiency/ or exp *vitamin supplementation/ or vitamin.ti,ab or exp *cyanocobalamin or cobalamin.ti,ab or cyanocobalamin.ti,ab or exp *anemia/ or an?emi*.ti,ab or exp *iron deficiency anemia/	2942372
2	exp folic acid/ or folic acid.mp or exp zinc/ or zinc.mp or exp copper/ or copper.mp or exp ceruloplasmin/ or ceruloplasmin.mp or hypocupr?emi*.mp or exp iron/ or exp iron overload/ or exp iron, dietary/ or exp iron metabolism disorders/ or exp iron compounds/ or iron.mp	535590	exp *folic acid/ or folic acid.ti,ab or exp *zinc/ or exp *zinc deficiency/ or exp *zinc urine level/ or zinc.ti,ab or exp *iron/ or exp *iron deficiency/ or iron.ti,ab or exp *copper/ or exp *copper deficiency/ or exp *copper blood level/ or copper.ti,ab or *exp *ceruloplasmin/ or exp *ceruloplasmin blood level/ or hypocupraemia.ti,ab	408920
3	exp weights and measures/ or weight.mp or exp body weight/ or exp body mass index/ or body mass.mp or exp body height/ or exp waist-	9128271	exp *weight/ or exp *body weight/ or exp *birth weight/ or exp *fetal weight/ or exp *ideal body weight/ or exp *gestational weight gain/ or exp *ideal	4164312

S1. Appendix 1. Search Strategy

	height ratio/ or height.mp or exp diabetes mellitus, type 2/ or exp overweight/ or exp obesity/ or exp diabetes mellitus, type 1/ or exp diabetes complications/ or diabetes.mp or exp waist circumference/ or circumference.mp or exp appetite/ or exp appetite depressants/ or exp appetite regulation/ or exp appetite stimulants/ or exp *weight loss/ or exp acute disease/ or acute disease.mp or BMI.mp or eat.mp or exp feeding behavior/ or feeding.mp or exp stress, psychological/ or exp stress, physiological/ or stress.mp or exp dietary fats/ or exp dietary fats, unsaturated/ or exp fats/ or exp fats, unsaturated/ or fat.mp or exp muscular atrophy/ or exp cachexia/ or exp muscular dystrophy, Duchenne/ or exp sarcopenia/ or musc* wasting.mp or musc* atrophy.mp or cachexia.mp or sarcopenia.mp or exp edema/ or oedema.mp or edema.mp or exp bariatrics/ or exp obesity,morbid/ or exp metabolic syndrome or metabol*.mp or exp bariatric surgery/ or bariatric.mp or exp hyperglycemia/ or hyperglyc?emi*.mp or exp hypoglycemia/ or hypoglyc?emi*.mp or exp insulin/ or exp insulin, short-acting/ or exp insulin, long-acting/ or insulin-like growth factor I/ or insulin-like growth factor II/ or exp insulin resistance/ or insulin.mp or exp skinfold thickness/ or exp anthropometry/ or exp body composition/ or thickness.mp or skinfold.mp or exp energy metabolism/ or exp energy intake/ or energy.mp		body weight/ or exp *low birth weight/ or exp *weight height ratio/ or exp *cuff weight/ or weight*.ti,ab or exp *height/ or exp *body height/ or exp *waist to height ratio/ or exp *weight height ratio/ or height.ti,ab or exp *abdominal circumference/ or exp *arm circumference/ or exp *chest circumference or exp *hip circumference/ or exp *neck circumference/ or exp *thigh circumference/ or exp * waist circumference/ or exp *head circumference/ or circumference*.ti,ab or exp *appetite/ or exp *appetite disorder/ or exp *appetite stimulant/ or exp *decreased appetite/ or exp *increased appetite/ or exp *sodium appetite/ or appetite.ti,ab or exp *acute disease or acute disease.ti,ab or exp *BMI chart/ or exp *body mass/ or exp *obesity/ or BMI.ti,ab or body mass.ti,ab or obesity.ti,ab or ability to eat.ti,ab or exp *feeding behaviour/ or exp *stress/ or exp *stress hormone/ or stress.ti,ab or exp *fat/ or exp *body fat/ or exp *fat mass/ or exp *fat free mass/ or exp *low fat diet/ or exp *pericardial fat/ or exp *perirenal fat/ or exp *subcutaneous fat/ or exp *subcutaneous fat disorder/ or fat.ti,ab or exp *muscle atrophy/ or muscle wasting.ti,ab or muscle atrophy.ti,ab or exp *edema/ or edema.ti,ab or oedema.ti,ab or exp *functional status/ or functional status.ti,ab or functional capacity.ti,ab or exp *bariatric surgery/ or bariatric.ti,ab or exp *morbid obesity/ or diabetes.ti,ab or exp *diabetes mellitus/ or exp *hyperglycemia/ or hyperglyc?emi*.ti,ab or exp *insulin/ or exp *insulin dependence/ or exp *insulin resistance/ or exp *long acting insulin/ or exp *short acting insulin/ or exp *pig insulin/ or insulin.ti,ab or exp *body distribution/ or exp *insulin sensitivity/ or skinfold thickness.ti,ab or triceps skinfold.ti,ab or exp *energy/ or exp *energy metabolism/ or exp *energy balance/ or exp *energy absorption/ or exp *energy expenditure/ or energy.ti,ab	
4	exp hyponatremia/ or hyponatr?emi*.mp or exp hyperkalemia/ or hyperkal?emi*.mp or exp methylmalonic acid/ or methylmalonic acid.mp or exp minerals/ or exp antioxidants/ or mineral.mp or exp albumins/ or albumin.mp or exp serum albumin/ or exp prealbumin/ or transthyretin.mp or exp transferrin/ or transferrin.mp or exp ferritins/ or ferritin.mp or exp	3689693	exp *hyponatremia/ or exp *hypernatremia/ or exp *hyperglycemia/ or exp *hypoglycemia/ or exp *insulin hypoglycemia/ or hypernatr?emi*.ti,ab or hyponatr?emi*.ti,ab or hyperglyc?emi*.ti,ab or hypoglyc?emi*.ti,ab or hyperkal?emi*.ti,ab or exp hyperkalemia/ or exp *methylmalonic acid/ or methylmalonic acid.ti,ab or exp	1115934

S1. Appendix 1. Search Strategy

	retinol-binding proteins/ or retinol.mp or RBP.mp or exp nitrogen/ or exp nitrogen compounds/ or exp reactive nitrogen species/ or exp nitrogen oxides/ or nitrogen.mp or exp trace elements/ or trace element.mp or exp selenium/ or selenium.mp or exp selenium compounds/ or exp selenium oxides/ or exp iodides/ or iodide.mp or skin sensitivity.mp or exp C-reactive protein/ or c-reactive protein.mp or CRP.mp or exp inflammation/ or exp inflammation mediators/ or exp neurogenic inflammation/ or inflammat*.mp or exp orosomucoid/ or orosomucoid.mp or alpha-1-acid glycoprotein.mp or exp lymphocyte count/ or exp leukocyte count/ or exp lymphocytes/ or lymphocyte.mp or exp hemoglobins/ or h?emoglobin.mp		*mineral/ or exp *mineral blood level/ or exp *mineral balance/ or exp *mineral deficiency/ or exp *mineral supplementation/ or exp *mineral intake/ or exp *mineral metabolism/ or mineral.ti,ab or exp *albumin/ or exp *serum albumin/ or exp *human albumin/ or exp *human serum albumin/ or exp *transthyretin/ or transthyretin.ti,ab or prealbumin.ti,ab or albumin.ti,ab or exp *transferrin/ or transferrin.ti,ab or exp *ferritin/ or exp *ferritin blood level/ or ferritin.ti,ab or exp *retinol-binding protein/ or retinol.ti,ab or RBP.ti,ab or exp *nitrogen/ or nitrogen.ti,ab or exp *nitrogen balance/ or exp *trace element/ or trace element.ti,ab or exp *selenium/ or exp *selenium deficiency/ or selenium.ti,ab or exp *iodide/ or iodide.ti,ab or exp *skin sensitivity/ or skin sensitivity.ti,ab or exp *C-reactive protein/ or c-reactive protein.ti,ab or CRP.ti,ab or exp *orosomucoid/ or orosomucoid.ti,ab or alpha-1 acid glycoprotein.ti,ab or exp *lymphocyte count/ or lymphocyte count.ti,ab or exp *hemoglobin/ or exp *glycosylated hemoglobin/ or h?emoglobin.ti,ab	
5	exp celiac disease/ or c?eliac disease.mp or exp glutens/ or gluten.mp or exp food hypersensitivity/ or exp intestinal mucosa/ or exp wheat hypersensitivity/ or eDL/ or exp cholesterol, LDL/ or exp cholesterol, VLDL/ or exp cholesterol ester storage disease/ or exp cholesterol esters/ or exp cholesterol oxidase/ or exp cholesterol, dietary/ or cholesterol.mp or exp hyperlipidemias/ or hyperlipid?emi*.mp or exp hypertension/ or hypertensi*.mp or exp gastroenterology/ or gastroenterolog*.mp or exp intestinal diseases/ or intestin*.mp or enteropath*.mp	1769635	exp *celiac disease/ or c?eliac disease.ti,ab or exp *gluten/ or gluten.ti,ab or exp *cholesterol/ or exp *cholesterol intake/ or exp *cholesterol level/ or exp *cholesterol blood level/ or exp *total cholesterol level/ or exp * high density lipoprotein cholesterol/ or exp *high density lipoprotein cholesterol level/ or exp * low density lipoprotein cholesterol/ or exp *low density lipoprotein cholesterol level/ or exp *very low density lipoprotein cholesterol/ or exp *hyperlipidemia/ or hyperlipid?emi*.ti,ab or exp *lipid/ or exp *lipid level/ or exp *lipid blood level/ or cholesterol.ti,ab or lipid.ti,ab or exp *gastroenterology/ or gastr*.ti,ab or exp *enteropathy/ or enteropathy.ti,ab or intestin*.ti,ab	2314643
6	exp nutrition assessment/ or nutrition* assessment.mp or prognostic nutritional index.mp or prognostic inflammatory index.mp or subjective global assessment.mp or Birmingham nutrition risk score.mp or nutrition risk classification.mp or malnutrition screening tool.mp or simple screening tool.mp or malnutrition universal screening tool.mp or nutritional risk screening 2002.mp or NRS 2002.mp or nutritional risk screening.mp or exp nutritional status/ or controlling nutritional status.mp or CONUT.mp	141188	exp *nutritional status/ or exp *nutritional assessment/ or prognostic nutritional index.ti,ab or prognostic inflammatory index.ti,ab or subjective global assessment.ti,ab or Birmingham nutrition risk score.ti,ab or nutrition risk classification.ti,ab or mini nutritional assessment.ti,ab or malnutrition screening tool.ti,ab or simple screening tool.ti,ab or full nutritional assessment.ti,ab or malnutrition universal screening tool.ti,ab or nutritional risk screening 2002.ti,ab or NRS 2002.ti,ab or short nutrition assessment questionnaire.ti,ab or	117942

	or Maastricht index.mp or nutritional risk index.mp or elderly nutritional indicators for geriatric malnutrition assessment.mp or ENIGMA.mp or swallowing.mp or exp deglutition/ or exp deglutition disorders/ or deglutition.mp or dysphagia.mp or speech language assessment.mp or SALT assessment.mp		controlling nutritional status.ti,ab or CONUT.ti,ab or Maastricht index.ti,ab or nutritional risk index.ti,ab or elderly nutritional indicators for geriatric malnutrition assessment.ti,ab or ENIGMA.ti,ab or exp *swallowing/ or swallow*.ti,ab or exp *dysphagia/ or dysphagia.ti,ab or exp *nasogastric tube/ or nasogastric.ti,ab or speech language assessment.ti,ab or SALT assessment.ti,ab	
~~7~~	~~3 or 5~~	~~2682508~~	~~3 or 5~~	
~~8~~	~~6 and 7~~	~~179059~~	~~6 and 7~~	
9	1 or 2 or 3 or 4 or 5 or 6	14775283	1 or 2 or 3 or 4 or 5 or 6	8315294

COMPARISON: N/A

AND

OUTCOME:

#	MEDLINE	Hits	EMBASE	Hits
1	exp injury severity score/ or exp severity of illness score/ or exp trauma severity indices/ or severity.mp or mJOA.mp or nurick.mp or exp spinal fusion/ or cooper myelopathy scale.mp or CMS.mp or exp outcome assessment/ or exp quality of life/ or prolo score.mp or European myelopathy score.mp or exp spinal stenosis/ or exp intervertebral disc displacement/ or EMS.mp or Copenhagen Neck Functional Disability Scale.mp or CNFDS.mp	832135	exp *disease severity/ or exp *disability severity/ or exp *pain severity/ or exp *severity of illness index/ or exp disease severity assessment/ or exp *injury severity/ or exp *fatigue severity scale/ or severity.ti,ab or exp *follow up/ or follow* up.ti,ab or exp *surgery/ or mJOA.ti,ab or nurick.ti,ab or exp *Nurick grade/ or cooper myelopathy scale.ti,ab or CMS.ti,ab or exp *quality of life/ or quality of life.ti,ab or prolo score.ti,ab or exp *spine fusion/ or exp *intervertebral disk hernia/ or disk hernia.ti,ab or European myelopathy score.ti,ab or exp *vertebral canal stenosis/ or EMS.ti,ab or Copenhagen Neck Functional Disability Scale.ti,ab or CNFDS.ti,ab or exp *pain assessment/ or disability index.ti,ab	4100509
2	neurological deficit.mp or outcome.mp or neurological benefit.mp or exp encephalomyelitis, autoimmune, experimental/ or enchephal*.mp or exp pain/ or exp eye pain/ or exp chronic pain/ or exp facial pain/ or exp back pain/ or exp low back pain/ or exp nociceptive pain/ or exp neck pain/ or exp musculoskeletal pain/ or exp pain management/ or exp pain measurement/ or exp pain perception/ or exp pain, intractable/ or exp pain, postoperative/ or exp pain, procedural/ or exp shoulder pain/ or exp visceral pain/ or exp pain insensitivity, congenital/ or exp pain, referred/ or pain.mp or neuropath*.mp or exp neuralgia/ or exp spinal cord injuries/ or exp reperfusion injury/ or exp stroke/	2752459	exp *neurological disease/ or neurological deficit.ti,ab or exp *pain/ or exp *neuropathic pain/ or exp *limb pain/ or exp *musculoskeletal pain/ or exp *visceral pain/ or exp *referred pain/ or exp *pleural pain/ or exp *postoperative pain/ or exp *procedural pain/ or exp *pain intensity or exp *pain measurement/ or exp *pain severity/ or exp *pain threshold/ or exp *posttraumatic pain/ or exp *spinal pain/ or exp *neck pain/ or exp *myofascial pain/ or exp *jaw pain/ or exp *inflammatory pain/ or exp *eye pain/ or exp face pain/ or exp *arm pain/ or exp *bone pain/ or exp *eyelid pain/ or exp *substernal pain/ or exp *wrist pain/ or exp *shoulder pain/ or pain.ti,ab or exp *neuropathy/ or exp *neuropathic pain/ or exp *brain	2852780

S1. Appendix 1. Search Strategy

			function/ or neurological function.ti,ab or neuropath*.ti,ab	
3	exp age of onset/ or onset.mp or exp treatment outcome/ or exp postoperative complications/ or outcome.mp or surgical results.mp or surgical management.mp or surgical success.mp or recovery.mp or exp vestibular function/ or exp ventricular function/ or exp respiratory function tests/ or exp recovery of function/ or exp pituitary function tests/ or exp liver function tests/ or exp kidney function tests/ or exp liver function tests/ or exp heart function tests/ or exp executive function/ or function.mp or neurological function.mp or exp patient readmission/ or readmission.mp or exp reoperation/ or reoperation.mp or exp hospitalization/ or hospitali?ation.mp or hospital*.mp	6499214	exp *adverse outcome/ or exp *adverse outcome pathway/ or exp *clinical outcome/ or exp *critical care outcome/ or exp *Glasgow outcome scale/ or exp *nursing outcome/ or exp *outcome assessment/ or exp *patient-reported outcome/ or exp *treatment outcome/ or outcome*.ti,ab or exp *late onset disorder/ or onset.ti,ab or surgical.ti,ab or exp *surgery/ or surgery.ti,ab or exp *anesthetic recovery/ or recovery.ti,ab or function.ti,ab or exp *function test/ or exp *motor function test/ or exp *muscle function/ or exp *musculoskeletal function/ or exp *parasympathetic function/ or exp *sympathetic function/ or exp *respiratory function/ or exp *spinal cord function/ or exp *vestibular function/ or exp *visual system function/ or exp *skin function/ or exp *urogenital system function and reproduction/ or exp *urinary tract function/ or exp *urethra function/ or exp *ureter function/ or exp *testis function/ or exp *sweat gland function/ or exp *sensorimotor function/ or exp *sexual function/ or exp *placental function/ or exp *pancreas function/ or exp *ovarian function/ or exp *ovary function/ or exp *nervous system function/ or exp *nerve function/ or exp *neuromuscular function/ or exp *mammary gland function/ or exp *liver function/ or exp *liver function test/ or exp *lacrimal gland function/ or exp *kidney function/ or exp *kidney function test/ or exp *intestine function/ or exp *intestine function disorder/ or exp *joint function/ or exp *hand function/ or exp *executive function/ or exp *executive function test/ or exp *endocrine function/ or exp *esophagus function/ or exp *cardiovascular function/ or exp *bladder function/ or exp *auditory system function/ or exp *adrenal cortex function/ or exp *adrenal function/ or exp *autonomic nervous system function/ or exp *cell function/ or exp *cognitive function test/ or exp *cardiopulmonary function/ or exp *brain function/ or exp *blood vessel function/ or exp *digestive system function disorder/ or exp *heart function/ or exp *mental function/ or exp *mental function assessment/ or exp *hospital readmission/ or readmission.ti,ab or exp *reoperation/ or reoperation.ti,ab or re-	2843340

S1. Appendix 1. Search Strategy

			operation.ti,ab or exp *hospitalization/ or hospitali?ation.ti,ab or hospital*ti,ab	
4	exp decompression, surgical/ or exp decompression sickness/ or exp spinal neoplasms/ or spinal cord decompression.mp or exp spinal cord compression/ or spinal cord compression.mp or spinal cord complications.mp or spinal cord surgery.mp	55521	exp *spinal cord decompression/ or exp *spinal cord compression/ or spinal cord decompression.ti,ab or spinal cord compression.ti,ab or exp *spinal cord surgery/ or spinal cord surgery.ti,ab	16991
~~5~~	~~2 or 4~~	~~483981~~	~~2 or 4~~	
~~6~~	~~1 or 2 or 4~~	~~1072061~~	~~1 or 2 or 4~~	
~~7~~	~~3 and 6~~	~~383400~~	~~3 and 6~~	
8	1 or 2 or 3 or 4	7476590	1 or 2 or 3 or 4	8030042

	MEDLINE- 1946 to Jan 28 2020	EMBASE 1988 to 2020 week 04
- Combining P7 and I9 and **O8** =	440	1183
- After modifications:	1088	2033
- After deduplicating:	820	2001
-		
- **After suggested modifications:**	3233**HITS**	3425 **HITS**
- **After duplicating across both databases**	**2466 HITS**	**3369 HITS**
- **TOTAL PAPERS:**	**5835**	
