# Supplementary material for: The Role of Nutrition in Degenerative Cervical Myelopathy: A Systematic Review
Source: Nutr Metab Insights. 2021 Oct 30;14:11786388211054664. doi: 10.1177/11786388211054664 (PMC8558601; doi:10.1177/11786388211054664)
Supplement: sj-pdf-2-nmi-10.1177_11786388211054664 – Supplemental material for The Role of Nutrition in Degenerative Cervical Myelopathy: A Systematic Review [file sj-pdf-2-nmi-10.1177_11786388211054664.pdf]

S4. Appendix 4.
Harvest Plot

This is a visual representation of the specific outcomes assessed in individual papers (refer to S5.Appendix 5 for bibliography).

Understanding the harvest plot:

The information is displayed in the following format:

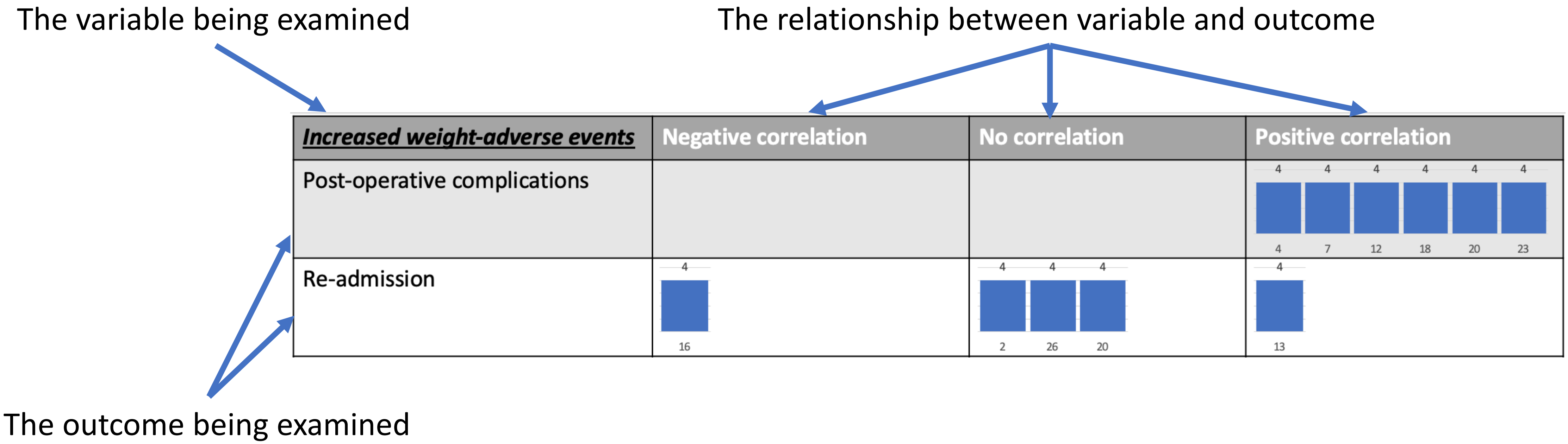

Understanding the harvest plot:

- **Each bar represents one paper.**

- The number at the base of each bar is a reference to the paper in the bibliography (Appendix 5)

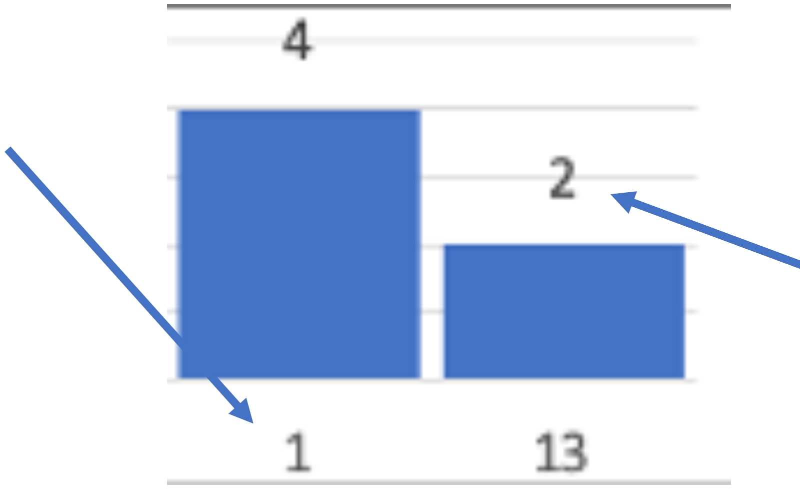

- The number at the top of the bar is the height.
- The height represents the quality of the paper based on if it was downgraded on any of 3 parameters: bias, imprecision, indirectness.

Bias, Imprecision, Indirectness	
	Height
If not graded down	4
If graded down on 1 parameter	3
If graded down on 2 parameters	2
If graded down on 3 parameters	1

Understanding the harvest plot: Example

Increased BMI -spinal cord	**Negative correlation**	**No correlation**	**Positive correlation**
Patient-reported outcomes	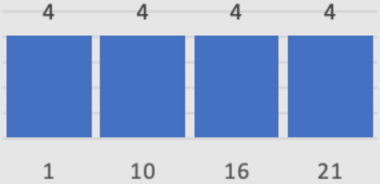	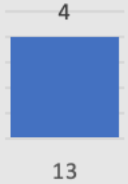	

This row shows papers looking at the relationship between increased BMI and patient reported outcomes (under the spinal cord biology category):

- 4 papers showed a negative correlation between these two variables,
- 1 paper showed no correlation,
- 0 papers showed a positive correlation.

S4. Appendix 4. Harvest Plot: Results

This is a visual representation of the specific outcomes assessed in individual papers (refer to S5.Appendix 5 for bibliography).

Increased BMI-adverse events	**Negative correlation**	**No correlation**	**Positive correlation**
Post-operative complications	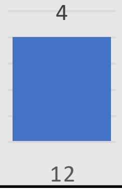	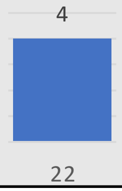	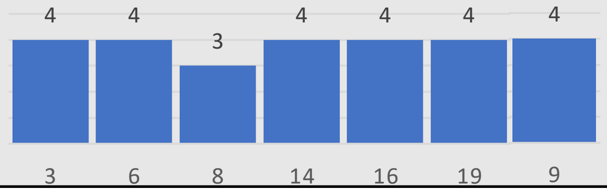
Mortality	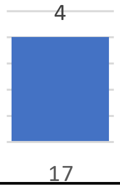	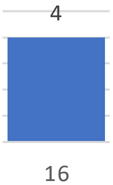	

<u>Increased BMI -spinal cord biology</u>	**Negative correlation**	**No correlation**	**Positive correlation**
Patient-reported outcomes	4 4 4 4<br>1 7 13 18	4<br>10	
Change in JOA score		4 4<br>13 25	
Functional independence measure efficiency			4<br>11
Reactive oxygen metabolites		4<br>21	

Increased BMI -spinal column biology	**Negative correlation**	**No correlation**	**Positive correlation**
Cervical alignment parameters			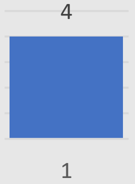
Modic changes			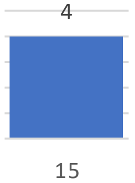
Loss of cervical lordosis		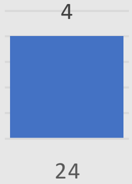	
Adjacent segment disease		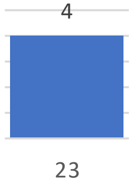	
Risk of cervical spondylosis		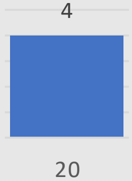	

Pre-operative weight loss- adverse events	**Negative correlation**	**No correlation**	**Positive correlation**
Post-operative complications			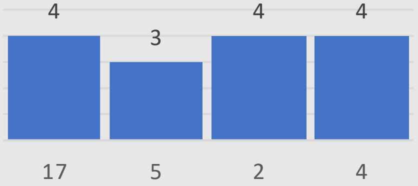
Mortality			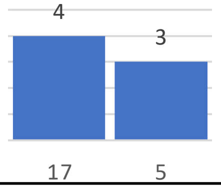

Malnutrition/electrolytes- Adverse events	**Negative correlation**	**No correlation**	**Positive correlation**
Post-operative complications		4 / 34	4 / 27, 4 / 29, 4 / 30, 4 / 28, 4 / 26, 4 / 32
Time for fusion			4 / 34

Malnutrition/electrolytes- Spinal cord biology	**Negative correlation**	**No correlation**	**Positive correlation**
Functional independence measure efficiency	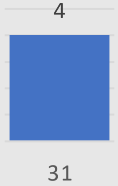		
Change in post-operative JOA score (RCT)	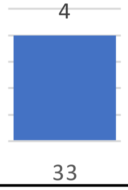		

<u>Vitamins/minerals-spinal cord biology</u>	**Negative correlation**	**No correlation**	**Positive correlation**
Upper extremity work capacity			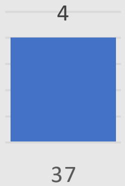
Change in post-operative JOA score (RCT)			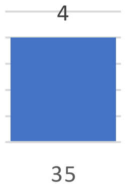
Duration of spinal cord injury			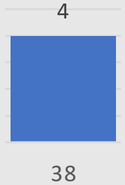

Vitamins/minerals-spinal column biology	**Negative correlation**	**No correlation**	**Positive correlation**
Risk of OPLL development	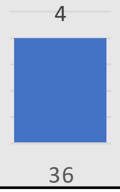		

Gastrointestinal health- Adverse events	**Negative correlation**	**No correlation**	**Positive correlation**
Post-operative complications			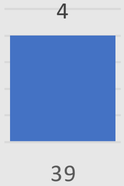

Gastrointestinal health-Spinal cord biology	**Negative correlation**	**No correlation**	**Positive correlation**
Patient-reported outcomes	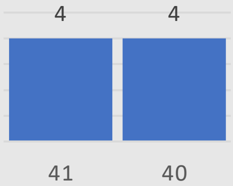		
Change in post-operative JOA score		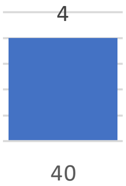	
